# Portrayal of the Human Resource Crisis and Accountability in Healthcare: A Qualitative Analysis of Ugandan Newspapers

**DOI:** 10.1371/journal.pone.0121766

**Published:** 2015-04-02

**Authors:** Silvia Wojczewski, Merlin Willcox, Vincent Mubangizi, Kathryn Hoffmann, Wim Peersman, Thomas Niederkrotenthaler, Silvia Natukunda, Samuel Maling, Manfred Maier, David Mant, Ruth Kutalek

**Affiliations:** 1 Department of General Practice and Family Medicine, Centre of Public Health, Medical University of Vienna, Vienna, Austria; 2 Nuffield Department of Primary Care Health Sciences, University of Oxford, Radcliffe Observatory Quarter, Oxford, United Kingdom; 3 Mbarara University of Science and Technology, Mbarara, Uganda; 4 Department of Family Medicine and Primary Healthcare, Ghent University, University Hospital, Ghent, Belgium; 5 Department of Social Medicine, Centre of Public Health, Medical University of Vienna, Vienna, Austria; University of North Carolina at Chapel Hill, UNITED STATES

## Abstract

**Background:**

Uganda is one of the 57 countries with a critical shortage of health workers. The aim of this study was to determine how the human resources and health service crisis was covered in Ugandan newspapers and, in particular, how the newspapers attributed accountability for problems in the health services.

**Methods:**

We collected all articles related to health workers and health services for the calendar year 2012 in the two largest national newspapers in Uganda (collection on daily basis) and in one local newspaper (collection on weekly basis). These articles were analysed qualitatively regarding the main themes covered and attribution of accountability.

**Results:**

The two more urban national newspapers published 229 articles on human resources and health services in Uganda (on average over two articles per week), whereas the local more rural newspaper published only a single article on this issue in the 12 month period. The majority of articles described problems in the health service without discussing accountability. The question of accountability is raised in only 46% of articles (106 articles). The responsibility of the government was discussed in 50 articles (21%), and negligence, corruption and misbehaviour by individual health workers was reported in 56 articles (25%). In the articles about corruption (n=35), 60% (21 articles) mention corruption by health workers and 40% (14 articles) mention corruption by government officials. Six articles defended the situation of health workers in Uganda.

**Conclusions:**

The coverage of accountability in the Ugandan newspapers surveyed is insufficient to generate informed debate on what political actions need to be taken to improve the crisis in health care and services. There exists not only an “inverse care law” but also an “inverse information law”: those sections of society with the greatest health needs and problems in accessing quality health care receive the least information about health services.

## Introduction

According to new data from the WHO there is a global shortage of 2.4 million medical doctors, nurses and midwives [1]. Uganda is one of the 57 priority countries that fell below the threshold of 2.3 doctors, nurses and midwives per 1000 people [2]. In 2012 only 58% of approved positions for health workers at all levels nationally were filled [3]. Shortage of medical staff has greatly compromised the delivery of quality health services in Uganda, and the lower the level of health care the higher the vacancy rate, which highlights the inverse care law in Africa [4–7]. Many Ugandan doctors and nurses migrate to Botswana, South Africa, Rwanda, UK, USA or Canada after completing their studies [8]. The latest statistics from the Uganda Medical and Dental Practitioners Council indicate that more than 2,000 or almost 50 per cent of the registered number of medical practitioners have left the country in the past 10 years [9–10]. One of the principal reasons for this shortage is the low level of pay and poor working conditions in government health facilities, which in turn are related to inadequate funding [4–7]. Uganda only spends 8% of GDP on health compared to the Abuja target of 15% [11].

Newspapers play an important role in engaging the public in political debate since they are a powerful and wide-spread source of information that can influence stakeholders. It is widely accepted that mass media provide one of several relevant sources and often a more valid source (particularly in low income countries) to inform about “out of the ordinary” events. Mass media are therefore, for example, also used for disease surveillance and other emergent public health threats [12]. Mass media can be influential in shaping and informing discourses about health and can reflect on issues embedded in health policy documents [13].

Therefore an analysis of the portrayal of health service delivery can be very useful to understand how the public is informed and opinions are formed about human resources in health [14–17].

Content analysis of newspapers is a research method which can provide important information about the political process [18–22]. In the Ugandan context, where internet coverage is limited, a qualitative content analysis can provide unique insight into what the public is being told about challenges of the human resource crisis in health [23]. Seale [17] notes that there are notably relatively few media studies focusing on health policy issues. Analyses of newspaper reports on the human resource crisis in sub-Saharan Africa are entirely lacking.

The aim of this study is to examine the public discourse of the human resource crisis in health in widely read newspapers in Uganda. The leading research questions for the qualitative content analysis are: How does the Ugandan press cover the shortage of human resources for health (HRH) and how is the media addressing the question of accountability for challenges of the resource crisis in health in Uganda?

## Material and Methods

### Design

This study took place as part of a larger EU-funded research project on human resources in primary health-care in Africa HURAPRIM (www.huraprim-project.eu) 2011–2015. For Uganda the team decided to do a newspaper analysis to find out how the media informed about the human resource crisis in health care delivery; a country with a severe shortage of human resources in health. We undertook a qualitative analysis of the content of three Ugandan newspapers, the two most-read national daily newspapers (published in English) and one regional newspaper (published weekly in the local language Runyankole). We identified articles about human resources and health care delivery published in 2012, undertook an in-depth qualitative analysis of the themes and perspectives taken in each article, adding a quantitative description of the frequency of each code identified and of the frequency of the type of article (news, opinion, editorial, letter, advertisement).

### Newspapers included

We included three newspapers that reflect diverse ownerships, political affiliations and were available in print. Although top newspapers in Uganda publish more and more online, a readership survey revealed that only a small proportion of newspaper readers visit news websites (higher socio-economic status, urban-based) [24]. The two English-language Ugandan daily newspapers with the widest circulation and readership in Uganda were included in the analysis—the *New Vision* (NV), a daily government-owned newspaper (80% of shares), and the *Daily Monitor* (DM), a daily and privately owned newspaper known to be critical to the government and often labelled as “enemy of the state” [25]. The *Daily Monitor* is published by the Nation Media Group of Kenya based in Nairobi and the *New Vision* is published by the New Vision Printing and Publishing Company Ltd (NVPPCL) that was set up in 1986 by president Yoveri Museveni. Nonetheless the NVPPCL Act of 1997 states that the *New Vision* remains independent. It was known to have a critical editorial board but lately has shown growing alignment with the government again [26, 25]. The Uganda All Media Products Survey (UAMPS) investigated that *New Vision* was the most widely-read newspaper, followed by the *Daily Monitor*. [26]. *New Vision* had a daily circulation of 34559 in 2012 and the *Daily Monitor* registered a circulation of 20673 copies daily [27]. To reflect diverse readerships we also included a local newspaper in Runyankole language, *Orumuri*, which is published once a week by the *New Vision* newsgroup and read by a more rural population. Runyankole is widely spoken in South West Uganda, with 2.3 million speakers [28].

All three newspapers are based in Kampala; however, the *Daily Monitor* has a new objective since 2011 to increase readership outside the capital and increased the number of journalists and transport outside of Kampala; the *Orumuri* is part of the New Vision Group strategy to publish in local languages to increase circulation in more rural parts of Uganda (in case of Orumuri the South Western part) [26].

The readers of the English language newspapers are by definition more educated and privileged than those who read newspapers in local languages [27]. That is why we included one regional newspaper in a local language. However, although the urban population is more likely to read a newspaper at least once a week, three-quarters of the readership of the *Daily Monitor* and the *New Vision* live in rural areas, which means that readership profiles should be well-covered by the inclusion of those three newspapers [24].

### Inclusion and exclusion criteria of articles

Two of the authors (SN and SM) systematically collected articles from these three newspapers from 1^st^ January to 31^st^ December 2012 on a daily/weekly basis. The inclusion criteria were selected by SN, SM, VM, RK and MW:

1. health workers:
  a. working conditions,
  b. payment,
  c. strike,
  d. service delivery
  e. other
2. lack of resources in health,
3. negligence in health,
4. corruption in health,
5. interventions in health,
6. best practice models in health

In order to achieve reliability of the data collection, three of the authors (SN, SM, VM) met on a monthly basis during 2012 to check the articles for the inclusion criteria and to exclude those who did not match. Articles were excluded post-collection when they were exclusively about health advice or about information on a certain disease and did not inform the debate on resources for health at all (e.g. articles that dealt exclusively with the nodding disease or with fistula).

After the data collection, all articles were scanned and made available to every research team member, to agree that inclusion criteria had been met and to facilitate subsequent analysis. As suggested by Muzyka, the team included all types of articles—news, letters, editorials and advertorials [18].

### Data analysis

#### Qualitative analysis

The scanned articles were imported into the CAQDAS (Computer Assisted Qualitative Data Analysis Software) atlas.ti. They were then analysed using the Hutter-Hennink qualitative research cycle [29]: All the articles were coded using an open/inductive approach [30–31]. In the first step of the qualitative analysis 20 articles were read in-depth and multi-coded openly by SW. Based on that a coding framework was defined which was applied to the other articles with additional open coding when new themes emerged (S1 Table). All the codes with quotations within one category were described and summarized with examples (by SW). Then, they were discussed with the co-authors.

All articles were re-read and analysed regarding attribution of accountability. Two of the authors (SW and RK) coded the articles deductively asking two specific questions of the material: Who is held accountable for challenges in the health sector in Uganda? All the already existing codes that had to do with accountability like “blaming government”, “Health policy_accountability“, “unethical behaviour of health workers” were again analysed to focus on attribution of accountability. Additionally, the articles about health facilities were also re-read focusing on attribution of accountability. The results were summarized by SW, reviewed and approved by the co-authors.

In all phases of analysis a constant and systematic reflection process checked and assured decisions about the coding scheme.

#### Quantitative analysis

The frequency of publication of articles was recorded for each newspaper, stratified by type (news, letter, editorial, opinion, advertorial). That means every article was attributed to the section it had originally appeared in the newspaper. Also, the frequency of each qualitative code and category was recorded using the quantitative features of the software atlas.ti.

#### Intercoder reliability

We calculated the percentage of agreement for each code to assess intercoder-reliability [32]. One additional researcher (EM) from Vienna coded twenty articles (10%; chosen randomly from the overall sample) independent of the other researchers. A percentage agreement for two coders (SW, EM) of more than 80% was achieved for the majority of the codes except for one code (i.e. “foreign aid” 75%, see Table 1).

**Table 1:** Intercoder test for two coders—Percent agreement.

Variable names	Percent agreement
HF_lack of equipment;	80
HF_lack of infrastructure;	80
HF_positive news	100
HP_accountability government;	80
HP_Government defends itself;	90
HP_government demanding/warning;	85
HP_government support_investments;	85
HP_political conflicts_power struggles;	85
HP_health budget;	90
HP_recruitment of HW	100
HP_good news from government;	100
HP_bad actions from gov;	80
HP_foreign aid;	75
HP_corruption;	95
HP_blaming government;	85
PAT_bad treatment;	100
PAT_agency;	100
HW_work/living conditions;	95
HW_unethical behaviour;	95
HW_shortage;	90
HW_positive news;	100
HW_payment_neg;	95
HW_emigration;	100
HW_education;	100
HW_difference urban/rural;	100
HW_demanding to;	100
HW_corruption;	95
HW_conflict;	100
HW_bad treatment;	100
HW_crime;	100
HW_empowerment	100

### Ethical approval

As the research leading to this manuscript was done with already published newspaper articles there was no ethical approval necessary.

## Results

A total of 287 potentially eligible articles about human resources and health were identified by the research team in Uganda, of which 230 articles matched inclusion and exclusion criteria for analysis by the authors who performed the content analysis.

### Frequency of publication of articles on health care delivery

Only one article was from the weekly regional newspaper *Orumuri*—the headline and key extracts are shown in Table 2.

**Table 2:** Single publication Orumuri (translated).

Single publication on health delivery in regional newspaper *Orumuri* in 2012
“**Erya Bwizibwera ritungire eza 5M**”
***Mbarara*: *Bwizibwera health centre got seats of 5 million*.** *Bwizibwera health centre four received seats costing 5 million Ugandan shillings from a kind person*, *Itungo Nathan*. *Itungo Nathan had visited the health centre in the past and found patients congested and scattered in the compound without where to seat while waiting to be attended to*. *The in-charge*, *Bob Bainomugisha*, *reported that the health centre which was constructed in the year 1940 has never had any new building constructed yet the number of clients has increased*. *This has led to over congestion of patients and also it does not have enough health workers*. *It had 50 health workers but at the present there are only 22 health workers and among them there is even no medical doctor yet there are supposed to be two medical doctors*. *He added on to say that due to lack of medical doctors they cannot carry out operations which make their theatre non-operational*. *(translation of the article from Orumuri*, 14.05.2012)

The other 229 articles appeared in the two national daily papers. The type of publication in the two national papers are summarised in Table 3.

**Table 3:** Type of publication in the two national papers.

	**Total**	**New Vision**		Daily Monitor	
	n	n	%	N	%
	229	104	45	125	55
**Type of article**					
News		81	78	98	78
Editorial		1	1	15	12
Sponsored advertorial		7	7	2	2
Letter or non-editorial opinion		15	14	10	8

### In-depth thematic analysis: health service delivery problems

The themes with health care delivery in Uganda that emerged recurrently in the articles in both newspapers could be categorised into three main themes—i) lack of access to care because of health worker shortages; ii) inadequate health facilities, including medicines; iii) poor behaviour and performance by health workers. There was reasonable concordance between the two national newspapers in the issues identified.

### Health worker shortages

Fifty-seven articles (25%) had as a theme the shortage of health personnel at all levels of care around the country (see Table 4). Most articles focused on the shortage of doctors but a lack of other skilled personnel was also reported. The country-wide shortage of midwives, skilled birth attendants or gynaecologists was often stressed (16 articles). Many articles described the more severe shortage in rural areas where often half of the posts in health care facilities were not filled and whole districts have no doctor. The few health workers left were said to be struggling to cope. It was reported that posts could not be filled due to low health budgets. Lack of infrastructure was cited as one major reason why it was so hard to retain or to attract health personnel. Several articles also mentioned the brain drain of doctors to other economic sectors (e.g. business) or to other countries (e.g. USA), arguing that this happened because of the poor pay in Uganda’s government health system. Other reasons cited were the fear to operate without having enough material, poor quality of care and economic reasons in general. Referring to numbers emigrating, one article reported: “*Uganda loses about 2*,*000 doctors and nurses every year*, *says the Permanent Secretary Ministry of Health*” (News, DM, 26.01.2012). The improvement strategies presented by the newspapers were better payment, better working conditions, training of quality staff (especially midwives are mentioned but also other critical staff, village health teams and traditional birth attendants to improve maternal health).

**Table 4:** Health staff shortages.

Statements made about staff shortages
“*Kapchorwa hospital*: *One doctor runs the show” (News*, *NV*, *29*.*01*.*12)*
“*Health sector short of 23*, *000 workers” (News*, *NV*, *25*.*09*.*12)*
“*We now have 17 health centres in Koboko district that would need over 200 health workers but on ground*, *less than 100 are battling to serve the people,” Dr XXX said.” (News*, *DM*, *04.10.12)*
“*Kalangala Islands lack efficient health services*. *There is a shortage of medical workers and health facilities” (News*, *DM*, *17.09.12)*

### Poor working conditions and health facilities

Twenty-six articles (11%) on the working conditions of health workers referred to negative aspects, including poor pay, no accommodation, no running water or electricity, especially in rural areas; staff shortages; extremely high workload; few drugs and little equipment to ensure treatment of patients and bad transportation infrastructure (see Table 5). Some articles stressed that it is difficult to attract and retain health personnel with such little pay (e.g. nurses, midwives)—it is in fact “*demoralizing*” (News, DM, 05.05.2012). Moreover, 67 articles (29%) reported on the lack of equipment and infrastructure in health facilities. Some articles reported that mothers have to deliver on the floor as there aren’t enough beds. Basic material such as drugs, vaccines and surgical gloves were said to be missing and many articles mentioned poor sanitation, lack of transport possibilities, no electricity, no water, lack of hospital beds and a general “*sorry state*” of facilities (News, DM, 17.09.2012) or a “*deplorable situation*” (News, NV, 06.05.2012). Many articles referred to a lack of ambulances or ambulances with mechanical problems or no fuel.

**Table 5:** Poor working conditions and facilities.

Statements made about poor working conditions and poor facilities
“*A health centre in Nebbi lacks delivery beds forcing mothers to use the floor” (News*, *NV*, *03.02.12)*
“*Yesterday*, *we lost two patients who could have survived if there was functioning equipment” (News*, *DM*, *31.10.12)*
“*Uganda’s health facilities are indeed in a very sorry state*. *[…] the situation is deplorable.” (Letter*, *NV*, *12.02.12)*
“*[Midwife:] My work station is 11km away from my home*. *[…] Sometimes I fail to make it to work due to lack of money for transport*. *The salary is not enough to sustain my livelihood.” (Op*, *NV*, *23.04.12)*
“*More than 50 per cent of the midwives in Uganda are stationed at rural health centres which do not even have accommodation*, *running water and power.” (News*, *NV*, *07.05.12)*

### Poor health worker performance and behaviour

Fifty-six articles (24%) focused on misbehaviour or poor performance of health workers (see Table 6); most often negligence, bribery and rudeness were mentioned (n = 42). Article themes included gross surgical mistakes, absenteeism, medical personnel being drunk, health workers refusing to treat without bribery, being rough to pregnant mothers and other patients. Most of the time those accusations come from patients who reported their experiences; persons that were frustrated because they had to wait the whole day without being attended to, were sent back home without medication, etc. Another important theme was about health workers demanding money from patients for supposedly free government services, such as paying for caesarean sections in government health facilities or for government supplied drugs. Unethical behaviour was especially often cited in relation to maternal health. A few articles also focused on health workers practicing without a licence or reported about illegal drug selling or illegal nursing schools (n = 14).

**Table 6:** Poor performance and behaviour of health workers.

Statements made about poor performance and behaviour of health workers
“*Besides this*, *all medics were extremely rude and arrogant to everyone.” (Letter*, *DM*, *24.09.12)*
“*Through the initiative [civil initiative VBC (Village Budget Club) of 20 men and women in a rural area] they have managed to identify problems with health centres such as unauthorized selling of drugs within the health unit*, *unqualified health volunteers demanding payment*, *drunkard midwives*, *all of which discourage women from accessing maternal health services.” (News*, *DM*, *08.12.12)*
“*I approached one of the nurses who told me that she would only help me if I gave her Shs 50,000 for a ‘soda’.” (Letter*, *DM*, *14.11.12)*
“*Though there is an increase in the number of donors […] unscrupulous health workers ask patients to buy blood.” (News*, *NV*, *24.02.12)*
“*A doctor’s negligence ruined her son’s life” (News*, *DM*, *25.02.12)*
“*Careless medics nearly cost Joy her life” (Ed*, *DM*, *24.05.12)*

### In-depth thematic analysis: accountability for health service delivery problems

Newspapers identified two main responsible entities for the health service delivery problems as raised in the articles. These were on the one hand the government that provides inadequate funding for health and on the other hand health workers and their behaviour. The findings for accountability are presented in detail below for the various dimensions of health service delivery problems identified.

### Accountability for shortage of health workers

In many cases (72%; 41 articles) the question of accountability was omitted from the articles about problems in human resource shortages. They tended to cite what is wrong and what is missing—for example, that patients are forced to buy basic medical treatment and have to pay for ambulances or they were left unattended due to lack of staff. There were also articles where inappropriate health worker behaviour (bad time management, moonlighting, absenteeism) was held responsible for staff shortages (n = 6). In some cases (n = 10) there were articles that placed direct demands to the government or held it accountable for shortage of staff. More often, government sources simply acknowledged the challenges in the health sector but shifted the responsibility onto something or somebody else (see Table 7).

**Table 7:** Accountability for shortage of health workers.

Statements attributing responsibility to central government
“*Government urged on maternal health” (News*, *NV*, *02.10.12)*
“*Recruit more health workers or face staffing crisis*, *MPs tell the government” (News*, *DM*, *05.09.12*)
“*Authorities in Mbarara district have appealed to the central government to recruit more health workers […] proper service delivery will remain a challenge unless government intervenes […]” (News*, *DM*, *21.03.12)*
“*Few or no health workers to serve the population demonstrates a failure to fulfil a legal obligation for which government must be held accountable […]” (News*, *DM*, *03.09.12)*
**Statements attributing responsibility to other stakeholders**
“*Ugly truth behind Lira hospital beauty […] the ministry is trying [to post health workers] but nobody wants to go upcountry” (News*, *NV*, *22.04.12)*
“*The health sector in Uganda has faced manpower crisis*. *[…] qualified health workers spend more time in their private clinics and attending workshops*, *leaving unqualified support staff” (News*, *DM*, *09.09.12)*
“*Nakaseke hospital lacks staff […] He [medical superintendent of Nakaseke hospital] said the Government has promised to construct 51 staff units*, *but the project has never taken off*. *The health ministry permanent secretary XXX said the district leaders mistreat doctors.” (News*, *NV*, *11.04.12)*
“*Shortage of staff hits Wakiso health units […] Although there are 28 workers for the health centres*, *only five were serving the islands*. *The health workers posted here work somewhere else.” (News*, *NV*, *14.09.12)*

### Accountability for lack of financing of health services

We found 14 articles that clearly linked the shortage of health workers in government health facilities to the inadequate funding for health. In general funding for health was an important issue in the newspapers in 2012 as the government planned budget cuts in the health sector in Uganda (34 articles about the health budget were found). Two main themes were identified: 1) political opposition to the budget cuts, with many Members of Parliament (MPs) demanding to cancel the cuts; 2) the disproportionate increase of the military budget in contrast to the budget for health. Interestingly when the pressure from the MPs led to the outcome that the health budget would not be cut, it appeared in the news as if health would get more money than before. Ultimately, MPs and public pressure only prevented the health budget from being cut; it was never increased at any point (see Table 8). That does not appear clearly in the newspapers. What does appear is how the government wants to allocate the budget: to the recruitment of health workers and the increase of doctors’ salaries, but only in one level of health centre (IV).

**Table 8:** Accountability for lack of funding.

Statements about accountability for lack of funding
“*Next financial year’s budget framework indicates that funding to the health sector might shrink*, *a move MPs threaten to oppose*. *[…] the reduction however*, *means*, *that the sector’s allocation will account for 8 per cent of the overall national budget which is way below the 15 per cent target in the Abuja declaration which Uganda ratified” (News*, *DM*, *07.05.12)*
“*MPs threatened to block the whole budget if government did not provide the needed Shs260b to resuscitate what they called a dead health sector*. *Part of it was needed to retain health workers currently on the pay roll (58 per cent staff establishment […])” (News*, *DM*, *17.09.12)*
“*I thank the government for its unquestionable vigilance when it comes to budgeting for security*. *I request our leaders to show equal or even more interest in the health sector because without health there can be no security.” (Ed*, *NV*, *24.10.12)*
“*The government yesterday bowed to pressure from the parliament to release Shs3.5billion for the recruitment of 6,172 health workers” (News*, *DM*, *28.11.12)*

### Accountability for poor state of health facilities

We found 12 articles (18%) where the government or the national medical store (NMS) were held accountable for the lack of facilities, equipment and medicines. In some of those poor management of the NMS was mentioned. Other articles reminded the government of its duties and commitments and sometimes blamed it for not taking action. They formulated demands to the government and in some cases made a direct connection between the mismanagement of funds by government officials and the poor facilities (see Table 9). In all other articles (82%; 55 articles) about the poor state of health facilities and lack of equipment either no one or the facility itself was held responsible.

**Table 9:** Accountability for poor facilities.

Statements about the accountability for poor facilities
“*Jinja RDC has ordered an investigation into Bugembe Health Centre after residents expressed dissatisfaction with services at the facility*. *[…] The directive followed revelations that the health centre […] lacks toilet facilities and is always short of drugs.” (News*, *DM*, *02.02.12)*
“*The President should help find the money to fix the health sector […] Any Ugandan who has visited the dilapidated government health facilities […] will be tempted to believe that life is not highly valued in Uganda*. *We can only conclude that for the President the wellbeing of ordinary Ugandans has never been and will never be a priority […].” (Letter*, *DM*, *21.09.12)*
“*At least 20 patients at Adjumani Hospital fail to get treatment every month because the medical facility lacks an ultrasound machine*. *[…] ‘This machine needs to be repared.’ The health ministry has been notified of the breakdown but has not made any formal response yet.” (Ed*, *DM*, *23.2.12)*
“*It has been a long known secret that most of the government ambulances are either idle*, *broken down*, *or being misused by government officials.” (Ed*, *DM*, *03.10.12)*

### Accountability for poor health worker behaviour and performance

The vast majority of the 56 articles dealing with negligence and misbehaviour of health workers implicitly or explicitly held individuals accountable *(“Health workers need to be reminded on their professional ethics” Letter*, *DM*, *14.11.12*). The articles mentioned health workers being sentenced for being absent, for being rude to patients or for gross negligence, having to pay fines. The articles also reported about patients that sued health workers or health facilities (see Table 10).

**Table 10:** Accountability for poor performance and behaviour.

Statements about accountability for poor performance and behaviour
“*The hospital administrator said he was not sure what happened during the time of the surgery*, *but that the error was not intended*. *Disciplinary action against the surgeons responsible will be effected” (News*, *DM*, *24.05.12)*
“*First Lady Janet Museveni has said negligence is contributing to the high death rates of women during pregnancy” (News*, *NV*, *28.03.12)*
“*A midwife at Ogoa health centre has paid a Shs150,000 fine to a woman she had allegedly roughed up.” (News*, *NV*, *28.01.12)*
“*Family sues hospital over death of mother*, *baby during delivery” (News*, *NV*, *24.04.12)*

Only six articles reflected on how politicians, management or patients deal with health personnel (see Table 11). These articles raised awareness about the difficulties that health workers face in Uganda, and the unfair way in which nurses are directly blamed by patients for the shortage of drugs and vaccines. Two opinion pieces strongly urged the Ugandan public not to victimize and blame health workers for all the problems in the health sector.

**Table 11:** Articles defending health workers.

Extracts from two articles urging the public not to blame health workers
“*They have not had vaccines for a year*. *Nursing officer:‘We only have the measles vaccine*. *Mothers who fail to access immunisation insult us*. *Unfortunately we were not alerted that there would be a shortage of drugs.’” (News*, *NV*, *13.10.12)*
“*He [a doctor] advises that maternal death audits look at whether systems are working well including the human resource challenges and not victimize individual health workers” (News*, *NV*, *17.10.12)*
“*One morning we are all going to wake up in Uganda and there won’t be any health worker anywhere to help us*. *This is because of the current allegations that health workers can’t be trusted and they need to be punished for their crimes*. *[…] The deeper issue is that Ugandan citizens are left to pay for things when the public health system should cover these costs*. *[…] When this doesn’t happen medics are forced to ask you to pay for supplies or if you’re lucky send you to a clinic that is stocked*. “*(Op*, *NV*, *25.09.12)*.

### Accountability for corruption

Most articles on accountability for health services went as far as reminding the government of its duties and commitments but few mentioned themes like misuse of funds or corruption (15%; 35 articles). In 21 articles (60%) about corruption and bribery, health workers were directly held accountable and condemned. They referred to health workers sentenced for stealing drugs and selling them in private medical stores, collecting money for free services and for bribery (see Table 12).

**Table 12:** Responsibility for corruption (1).

Corruption of health workers
“*Koboko medics jailed for stealing drugs” (News*, *DM*, *17.10.12)*
“*Alebtong resident district commissioner (RDC) has cautioned health workers against collecting money from patients*, *saying it discourages people from seeking help from health units.” (News*, *NV*, *10.04.2012)*
“*Health workers cited in bribery*. *Health monitor unit indicates that patients in Fort Portal have to bribe to receive treatment.” (News*, *DM*, *04.09.12)*
“*A health worker from Butuntumula health centre III […] is reportedly on the run after government drugs were recovered from his drug shop.” (News*, *DM*, *07.05.12)*
“*My prayer is that health workers revise their professional ethics*. *[…] Above all bribery should have no place in our hospital.” (Letter*, *DM*, *04.11.12)*

Fourteen articles (40%) were identified that directly wrote about corruption by politicians and the misuse of funds as a reason for the bad state of the health sector; most addressed the issue of corruption only in a side-remark. One debate about mismanagement of health funds was about the prosecution of health officials over the misuse of GAVI funds (*The Global Alliance for Vaccines and Immunisation*) (5 articles). Only one article was identified where the shortage of health workers was linked to corruption by high-level politicians and insufficient funding for health (see Table 13).

**Table 13:** Responsibility for corruption (2).

Corruption in the government
“*Health ministry officials pinned over GAVI funds […] Over Sh3b meant for immunization was reportedly misused by individuals*, *Government agencies and civil society organization*. *As a result Global Fund stopped funding Uganda […].” (News*, *NV*, *24.10.12)*
“*When health funds or medicines do not reach their destination*, *the deaths that result are on the hands of the misappropriating officials*. *Corruption is a silent remote mass murderer […]” (Op*, *NV*, *26.04.12)*
“*Bribery claims rock health budget crisis*. *Several ruling party MPs yesterday went back on their vow to ensure Shs260 billion to fix the health sector was included in the budget*, *an apparent change of heart some of their colleagues said was secured through suspected bribery*. *[…] Museveni was giving them allowances and told them that they should pass the budget the way it is*. *This is corruption*. […] *Although Ministry of Health had tabled a request Shs260b needed to motivate and recruit more health workers*, *the Minister for Health yesterday reduced the need to only Shs49.5b.” (News*, *DM*, *26.09.12)*

## Discussion

This study with Ugandan newspapers is the first qualitative media analysis regarding human resources and health services in Africa. Although the national newspapers frequently write about the problems of health service delivery, they very often criticise health workers misbehaviour without giving enough background for the severe human resource shortage in Uganda and thus fail to show the hard conditions government health workers are facing. Moreover, health service issues are much more covered in national urban newspapers than in a local more rural newspaper, which suggests that there may be an “inverse information law”.

### Strengths and limitations of the study

The strengths of our approach are that the selection of articles was systematic and comprehensive and that their analysis was qualitative complemented by a quantitative description of the frequency of the codes. To be able to include different views we included one newspaper that is known to be privately owned and the other to be government-owned. These are both in English which is one of the official languages in Uganda but we also included one regional newspaper in a local language, Runyankole. The qualitative analysis complements statistical data on the shortage of health workers globally [32]. We got an in-depth insight into the themes and a lot of different stakeholders’ views as we included news and opinion pieces. Qualitative media content analyses are still rare and our research adds to fill that methods gap.

Limitations are that the search was limited to one year, to one type of media, and to three printed newspapers. One reason for choosing only print media was that in 2012 only 14.7% of the population in Uganda had internet access [33–34], and even those are disturbed by slow connections and power cuts, so relatively few people read online versions of newspapers and each paper copy is read by several people. Furthermore, we only included one regional weekly newspaper in a local language. One other limitation is the possible bias of the newspapers. It is true that the material which is provided by the mass media for “reality construction” is generally selective and does not comprehensively reflect social realities [35]. This is particularly due to their focus on unique, and “out of the ordinary” issues [36] which is at least partially at odds with the public health focus on frequent and “ordinary” issues. [37]. It is widely accepted that mass media influence political decision making and form public opinion on specific topics of societal relevance [38]. This makes an analysis of media content relevant to any politically sensitive issue.

The inclusion of several media sources (3 different popular widely read media sources in English and Runyankole) in our study is likely to reflect a broader and less-biased spectrum of opinions and issues as compared to other sources which are mandated or funded by the government, like the Ugandan Medical and Dental Practitioners Council.

The local weekly newspaper *Orumuri* is known to have a wide circulation area especially in western Uganda [39, 40]. The two included English newspapers have the widest circulation and readership in Uganda. A BBC report [26] states that newspapers in Uganda play a critical role and set the agenda for national conversations. Both reports point out that radio stations review the Kampala national press (to which New Vision and the Daily Monitor belong) and that stories are discussed on air with listeners who call in. Thus, the selected newspapers certainly are influential opinion-formers in Uganda.

## Principal Findings

Each of the two national newspapers had on average more than two stories per week on human resources and health service delivery. In contrast, the local weekly Runyankole newspaper had only one article in the whole year regarding health service delivery. That article just cites the bad state of a health facility and inadequate staffing without exploring the causes. This is paradoxical, because the readers of the local newspaper are probably at greater risk of health problems than the more educated readers of the English language national newspapers. As the *Orumuri* is written in the local language Runyankole and the circulation area is the more rural and western part of Uganda, the newspaper very likely is read by people with a) a lower educational background b) a lower socio-economic status and c) by people hit harder by the human resource crisis in health, as they are more dependent on good affordable public health care than richer stratums that can afford private health care. The fact that we found only one article about human resources for health suggests that the people who need the most information about the rights and wrongs of health service delivery in Uganda receive the least. Thus there seems to be not only an “inverse care law” [41] but also an “inverse information law”. Those with the greatest need for information on health services receive the least information. The BBC media action report from 2012 supports that result as it notes that more local media in Uganda are more and more subjected to political censorship [26].

The fact that we found a considerable number of articles in the two main national newspapers shows the high relevance of the theme “human resources in health” in the public opinion in Uganda. Both daily newspapers mentioned explicitly what the problems in public health care were, covering rural and urban areas throughout the country. That suggests that regular newspaper readers can get an impression of health care situations around the country. Also both newspapers described views from a variety of stakeholders and published personal patient stories, editorials and letters about problems in health service delivery in Uganda. That finding suggests that both newspapers no matter if privately or government-owned can well exercise their press freedom rights.

The commonest themes were poor health facilities and the considerable shortage of staff but causes, consequences and accountability for this situation were rarely mentioned. The themes of corruption and mismanagement of funds and medicines sometimes appeared in the articles (n = 35/230). Of those, 60% (21 articles) mention corruption by health workers and only 40% (14 articles) mention corruption by government officials (see Table 12 and Table 13).

Bribery and unofficial payments of health workers in Uganda are indeed a serious issue. However, there are few empirical studies that deal with this important theme [42–45]. They underline that the root causes of bribery and unofficial payments in health care are low payment of health workers and faulty implementation of health care reforms and that corruption happens at all levels of health care—from the worker to the government level. Corruption happens mostly in countries “where there is no framework of good government [and] no rule of law” [46]. As anti-corruption strategies the studies identify transparency, accountability, higher wages and benefits for health workers. It would be important that newspapers include that kind of information in health reporting, too.

It is positive that newspapers report about corruption in general as it is of high public interest; the newspapers can show how people are affected by corruption and who is to blame. However, the focus on reporting about corruption lies on low-level corruption; little is said or done about corruption on higher government level that is responsible for the diversion of vast funds originally foreseen for the health sector [45–47]. Both newspapers did not report much about high-level corruption. However, there was a difference between the two newspapers regarding the section they reported about it. The *Daily Monitor* reported about corruption mainly in the news and editorial section; the *New Vision* mainly in the Opinion/Letters section. Any sound and valid indication of a politically sensitive issue such as corruption requires an adequate, independent control system. In Uganda, anti-corruption mechanisms such as the Anti-Corruption Court, the Office of the Inspectorate of Government, the Auditor General or the Uganda Medical and Dental Practitioners Council (where cases of professional misbehavior can be reported) are available. The Inspectorate of Government reports corruption cases to the parliament every year, including corruption in the health sector. However, while the institutions prosecute low-level corruption rather well, they have been ineffective in curbing grand scale corruption. Since 1986, two Ministers have been convicted for corruption-related offenses. In one case, the president publicly offered to pay for the defendant’s legal fees: a former Minister for Health was convicted in 2013 (and then acquitted on appeal) for embezzling 210 million Ugandan shillings (around $80,000) from Global Fund grants while many more government officials were accused of misappropriating funds, according to a report from Human Rights Watch [47]. It seems that neither the media nor anti-corruption institutions in Uganda focus on revealing or prosecuting high-level corruption.

We think that newspapers should show a more balanced picture when reporting about corruption and misbehaviour of health workers. That would make it easier for the reader to disentangle the complex structure of corruption in government health-care. The one-sided picture of bribery and unofficial payments in the Ugandan newspapers reinforces misinformation and mistrust between patients and health workers and hides the more structural policy issues behind corruption.

In the articles we found that patients tended to blame the health workers, even when the problems were not their fault. Even the best-intentioned health workers are at risk of becoming demotivated when they lack the tools and supplies to do their job, for reasons beyond their control. Surprisingly, from the few articles that defended the case of health workers (n = 6), only one was from the *Daily Monitor*. The articles point out that health worker misbehaviour is part of a bigger challenge of human resources for health. It seems that more articles could be issued about that (Table 11).

In the case of the health budget debate it is surprising that the newspapers did not pursue the question of why so many MPs that fought for more health funding in the beginning stopped doing so after speaking to the president. Why did the political opposition stop advocating for more health funding? None of the two newspapers asked that question. However in general, the *Daily Monitor* reported more than the *New Vision* about the plans of the government to cut the health budget in 2012, especially in the “news” section. *New Vision* concentrated more on the positive part of the budget debate, where the government decided to increase doctor’s salaries; the *Daily Monitor* focused more on the topic that the government wanted to cut health budget and what political conflict that created.

A BBC media report [26] found out that the *New Vision* used to be fairly independent and critical but has since a few years been growingly aligned with the government. The *Daily Monitor* together with the *Crusader*, the *Observer* and *Independent* on the contrary are classified as forming the independent “vanguard of a lively national press”. That can partly explain why we found more articles in the opposition newspaper *Daily Monitor* on the government’s accountability than in the government-owned *New Vision*.

Although there is a vast number of radio stations in Uganda (over 200) and some 48 newspapers are published [48] the press in Uganda is not completely free [26]. In the World Press Freedom Index from 2014, Uganda is listed 110/180 [23]. Until the 1990s the media was under state control with the liberalisation of the media following in 1993. In 2013 the offices of the *Daily Monitor* were shut down by the police for 11 days after publishing a letter to which the President objected and some cases are known where journalists were imprisoned or physically attacked [49,50]. Therefore even opposition newspapers must be careful about criticising the government. The report from the Friedrich-Ebert-Stiftung on the other side concludes that press freedom has improved in many regards over the last years and the BBC report from 2012 states that the press is free especially the urban, middle class- oriented media, which would include the *Daily Monitor* and the *New Vision* [26,27].

Overall, we think that newspapers could give more background on the severe human resource crisis in health when writing about unethical behaviour of health workers and they could show a more balanced picture about accountability of individual health workers and accountability of the government.

### Unanswered questions and future research

It would be interesting to study whether other local newspapers in Uganda also have the same dearth of articles on health services as *Orumuri*, and to explore interventions to improve coverage of these issues in local media. It would also be interesting to study whether a more supportive attitude towards health workers, highlighting those who do a good job rather than simply criticising misbehaviour overall, would help to improve the motivation of health workers and to positively influence any misbehaviour.

There is, however, a broader question around democracy and political debate. One of the main roles of media should be to engage the public in political debate and to hold politicians to account. Meaningful improvements are dependent on good governance which will only be achieved when the public, including the media, addresses issues of accountability for the state of the health service and the lack of health workers in the country.

## Conclusions

The coverage of accountability in the Ugandan newspapers surveyed is insufficient to generate informed debate on what political actions need to be taken to improve health services. Although the national newspapers often write about the problems of health service delivery, they too often criticise health workers misbehaviour without giving the background of the severe human resource shortage in Uganda and thus fail to show the hard conditions government health workers are faced with. We found that there was too little information about the responsibilities and accountability of the government to provide adequate health to its population. It is unlikely that the current state of reporting about the human resource crisis in health will contribute to the improvement of the situation. Health service issues were much more covered in national newspapers than in a local newspaper, which suggests that along with an “inverse care law” there might be an “inverse information law”. The more rural, socio-economically vulnerable readership of the local newspaper receives the least information about the human resource crisis in health although they would need it the most. In order to improve the situation in the long run, it is important that the media start showing a more balanced picture between responsibility of health workers and responsibility of government. This would help the broader public to identify workable potential solutions to the current resource crisis in health care.

## Supporting Information

S1 Table Code family/code: names and descriptions. S1 Codes: names and descriptions.
(DOCX)
