# Supplementary material for: Portrayal of the Human Resource Crisis and Accountability in Healthcare: A Qualitative Analysis of Ugandan Newspapers
Source: PLoS One. 2015 Apr 2;10(4):e0121766. doi: 10.1371/journal.pone.0121766 (PMC4383446; doi:10.1371/journal.pone.0121766)
Supplement: S1 Table — S1 Codes: names and descriptions. (DOCX) [file pone.0121766.s001.docx]

**Supporting information**

**S1 Table.**

**Code family/code: names and descriptions**

S1 Codes: names and descriptions

| **Name of Code family (Category)/code** | **Code family /code description (the codes were attributed if the theme was mentioned, also if it was mentioned only briefly): Article mentions…** |
| --- | --- |
| *Code family*  *Health facilities (HF)*  *Codes:* | *Equipment and infrastructure lacking/missing in health care provision in Uganda* |
| HF_lack of equipment; | lack of blood, drugs, not functioning equipment |
| HF_lack of infrastructure; | lack of infrastructure or bad conditions of health facilities  lack of ambulances, poor roads, no beds, poor accommodation for staff,  lack of money for maintenance |
| HF_positive news | Good news about health facilities: new constructions, investments, inauguration of health facilities |
| *Code Family:*  *Health policy (HP)*  *Codes:* | *National, regional politics in health care in Uganda* |
| HP_accountability government; | reminding the government on its commitments,  showing were government did not meet its obligations  Formulating direct or indirect demands to the government; articles mentioning demands of parliament, organizations or single persons towards government. |
| HP_Government defends itself; | Government defending itself, naming reasons when lack of personnel, infrastructure or medical equipment is mentioned |
| HP_government demanding/warning; | Government demanding, urging or warning public or government officials or organisations or other regarding health issues |
| HP_government support_investments; | any kind of investments in health care sector:  government supporting construction/renovation of health facilities, buying medical equipment, or offering better salaries or offering cooperation with Unions (for preventing strikes) |
| HP_political conflicts_power struggles; | Internal conflicts within political representatives, dismissals, but also conflicts with Unions or between government and the opposition  unclear division of tasks in political administration |
| HP_health budget; | health budget (cuts/increases; what happens with the budget); demands from stakeholders (e.g. parliament, union, individuals) to have a health budget increase |
| HP_recruitment of HW | Positive news of recruitment of health personnel; demands from different stakeholders to the government to recruit health workers; calls to apply for a special health cadre in an organisation or government |
| HP_good news from government; | showing positive actions from the government  about investments in health (recruitment, construction and equipment provision) or about offering better salaries. Others show the government cooperating with Unions |
| HP_bad actions from gov; | Negative actions from government destructing health facilities, budget cuts for health unities, not paying health workers, negligence of political representatives (mentions health ministry, individual government officials, government) |
| HP_foreign aid; | foreign aid regarding health funding, which country or which international organization donated or funded a project or a construction for health |
| HP_corruption; | openly writing about corruption cases within the political cadres to do with the health sector of Uganda. |
| HP_blaming government; | blaming government or certain government officials for problems in health sector;  showing wrong priorities of health politics in Uganda;  Words like “accountability, failure, accusing, prosecution, suspension, misuse, blame, below its mandate, abuse, mismanagement” come up in those articles pointing out that the government is not fulfilling its mandate in the health sector and are responsible for negative developments |
| *Code family:*  *Health workers (HW)/ patients (PAT)*  *Codes:* | *Mentioning health workers or patients* |
| PAT_bad treatment; | patients left unattended or treated badly (had to pay for services that are supposed to be free) or given false medication, or surgery went wrong or cannot attend health facility because of bad infrastructure/logistical problems |
| PAT_agency; | stories about lives of patients or family of patient; patients or family who take actions on their right to health |
| HW_work/living conditions; | Mentions something about working and /or living conditions of health personnel |
| HW_unethical behaviour; | unethical behavior from health workers going from bad treatment of the patients to more severe crimes like practicing illegally;  negligence, absenteeism, bribery, being rough; drug misuse or misuse of other equipment |
| HW_shortage; | Shortage of health personnel in all kinds of health centers around the country. Shortage of all kind of health cadres from skilled birth attendant to specialists. |
| HW_positive news; | Graduations, pay rise, recruitment of health workers, good performances from health workers |
| HW_payment_neg; | bad news about payment of health personnel |
| HW_emigration; | Emigration of health personnel internationally or to other economic sectors or from rural to urban |
| HW_education; | News about graduations of health workers, demands to train health cadres, demands for continuous training of health workers |
| HW_difference urban/rural; | Differences in working and living conditions in rural and urban areas |
| HW_demanding to; | health workers demanding better work conditions |
| HW_corruption; | misuse of funds from HW; criminalization of health workers; mentioning openly bribery, corruption from health workers |
| HW_conflict; | Conflicts between HW groups or between HW and politics |
| HW_bad treatment; | Defending hw/ unfair treatment of health workers  by the management, politics or by patients, communities |
| HW_crime; | Illegal health facilities or illegally operating health workers; crimes committed by health workers or health organizations or by persons acting as health workers illegally (quaks) |
| HW­_empowerment | health cadres on strike, demanding higher salary and better working conditions, or individual health workers openly speaking about their situation |
